# Isolation, Biochemical Characterisation and Identification of Thermotolerant and Cellulolytic *Paenibacillus lactis* and *Bacillus licheniformis*

**DOI:** 10.17113/ftb.59.03.21.7096

**Published:** 2021-09

**Authors:** Krzysztof Makowski, Martyna Leszczewicz, Natalia Broncel, Lidia Lipińska-Zubrycka, Adrian Głębski, Piotr Komorowski, Bogdan Walkowiak

**Affiliations:** 1 Industrial Biotechnology Laboratory, Bionanopark Ltd., Dubois 114/116, 93-465 Lodz, Poland; 2 Biotechnika, Tymienieckiego 25, 90-350 Lodz, Poland; 3 Institute of Genetics and Biotechnology, Faculty of Biology, University of Warsaw, Miecznikowa 1, 02-096 Warsaw, Poland; 4 Molecular and Nanostructural Biophysics Laboratory, Bionanopark Ltd., Dubois 114/116, 93-465 Lodz, Poland; 5 Institute of Materials Science and Engineering, Lodz University of Technology, Stefanowskiego 1/15, 90-537 Lodz, Poland

**Keywords:** *Paenibacillus lactis*, *Bacillus licheniformis*, cellulolytic activity, thermotolerant bacteria, carboxymethylcellulose (CMC), BioLector® microbioreactor

## Abstract

**Research background:**

Cellulose is an ingredient of waste materials that can be converted to other valuable substances. This is possible provided that the polymer molecule is degraded to smaller particles and used as a carbon source by microorganisms. Because of the frequently applied methods of pretreatment of lignocellulosic materials, the cellulases derived from thermophilic microorganisms are particularly desirable.

**Experimental approach:**

We were looking for cellulolytic microorganisms able to grow at 50 °C and we described their morphological features and biochemical characteristics based on carboxymethyl cellulase (CMCase) activity and the API® ZYM system. The growth curves during incubation at 50 °C were examined using the BioLector® microbioreactor.

**Results and conclusions:**

Forty bacterial strains were isolated from fermenting hay, geothermal karst spring, hot spring and geothermal pond at 50 °C. The vast majority of the bacteria were Gram-positive and rod-shaped with the maximum growth temperature of at least 50 °C. We also demonstrated a large diversity of biochemical characteristics among the microorganisms. The CMCase activity was confirmed in 27 strains. Hydrolysis capacities were significant in bacterial strains: BBLN1, BSO6, BSO10, BSO13 and BSO14, and reached 2.74, 1.62, 1.30, 1.38 and 8.02 respectively. Rapid and stable growth was observed, among others, for BBLN1, BSO10, BSO13 and BSO14. The strains fulfilled the selection conditions and were identified based on the 16S rDNA sequences. BBLN1, BSO10, BSO13 were classified as *Bacillus licheniformis*, whereas BSO14 as *Paenibacillus lactis*.

**Novelty and scientific contribution:**

We described cellulolytic activity and biochemical characteristics of many bacteria isolated from hot environments. We are also the first to report the cellulolytic activity of thermotolerant *P. lactis*. Described strains can be a source of new thermostable cellulases, which are extremely desirable in various branches of circular bioeconomy.

## INTRODUCTION

Due to the constantly growing human population, the demand for energy increases, while oil deposits are gradually depleted. Therefore, the latest research is focusing on the exploration of alternative energy sources. Among them, the biofuels produced from renewable materials are the most promising. Raw materials such as biogas and bioethanol can become an inexpensive approach to prevent the development of problems related to environmental protection (*1*, *2*). It is estimated that in 2016 the use of biofuels reduced CO_2_ (equivalent greenhouse gas) emission by 43.5 million metric tonnes, which corresponds to removing 9.3 million cars from the roads (*3*). Moreover, bioethanol production has a positive effect on the economy, supporting local agricultural sectors and rural communities. It also increases energy security by reducing dependence on crude petroleum and diversifying energy supplies (*4*).

For biofuel production, energy crops such as corn, rice, rapeseed, sorghum, grass, as well as various industrial and agricultural wastes are usually used (*5*). First generation bioethanol is produced from raw materials rich in starch (*e.g.* wheat, barley, corn, potato, cassava) and sucrose (*e.g.* sugar beet, sugarcane). Nevertheless, controversies related to the use of food feedstocks increased the interest in the conversion of waste materials. As a result, second generation fuels were obtained. Currently, due to wide distribution and availability, lignocellulose is one of the most important substrates applied in biofuel production. Lignocellulosic biomass and waste crops can be a substrate for production of respectively 442 and 491 billion litres of bioethanol (*6*, *7*).

Lignocellulosic mass is present mainly in leaves, stems and shoots. It consists of three main fractions: cellulose, hemicellulose and lignin. The main component of the waste lignocellulosic biomass is cellulose (30-50%). It is a non-branched polysaccharide that contains from 3000 to 14 000 d-glucose residues, linked by β-1,4-glycosidic bonds (*8*). Hemicellulose (25-50%) is a heterogeneous group of polysaccharides and its derivatives contain monosaccharides such as hexose (glucose, mannose and galactose), pentose (xylose, rhamnose and arabinose) and uronic acid. The subunits are linked by β-glycosidic bonds, forming branched chains. Lignin (10-30%) is a condensation polymer consisting of aromatic phenolic alcohol derivatives such as coniferyl, sinapyl and coumaryl alcohols, which are cross-linked with carbon-carbon (C-C) ether and covalent bonds (*9*, *10*).

Despite many efforts, the conversion of lignocellulosic biomass to bioproducts is still expensive. The biofuel production process requires not only the transport of biomass to a suitable place but also physical or chemical pretreatment followed by hydrolysis. Efficient biosynthesis can be obtained only if the costs related to the above operations are reduced (*11*). Moreover, fermentation should be improved to achieve more efficient utilization of the lignocellulosic biomass, higher ethanol yield, productivity and concentration in the fermentation medium (*12*, *13*).

Thermophilic microorganisms are widespread in diverse environments such as soil, geysers, hot springs, compost systems, hydrothermal vents and heated beach sediments (*14*). They can be divided into three groups: moderate thermophiles, extreme thermophiles and hyperthermophiles with the optimal growth temperature in the range of 50-60 °C, 60-80 °C and 80-110 °C respectively (*15*). The thermophiles are a source of valuable enzymes that can be applied in various industries. It is worth remembering that the solubility of reagents and products increases at higher temperature. Therefore, chemical reactions can be faster and it is possible to use a decreased amount of enzyme. Moreover, in the case of biotechnological processes, the high temperature reduces the risk of microbial contamination. Thermophilic enzymes usually exhibit higher stability and flexibility, so their use facilitates the recovery of ethanol and other volatile products. These benefits encourage the application of thermostable enzymes for the hydrolysis of lignocellulosic material (*16*).

Both fungi and bacteria can produce a wide range of enzymes capable of breaking down the lignocellulosic biomass (*17*). For complete and effective cellulose breakdown three components are necessary. First, 1,4-β-d-glucan glucohydrolase [EC 3.2.1.4] (endoglucanase), which breaks down cellulose randomly to shorter fragments such as cellobiose and glucose; second, 1,4-β-d-glucan cellobiohydrolase [EC 3.2.1.91] (an exoglucanase), which works on the outer parts of the cellulose molecule and releases cellobiose from reducing or non-reducing ends, and third, β-d-glucoside glucohydrolase [EC 3.2.1.21] (β-glucosidase), which catalyzes the hydrolysis of the terminal, non-reducing β-d-glucosyl residues with the release of glucose (*18*, *19*).

Due to easier extraction and purification, fungi are most commonly used for cellulolytic enzyme production (*20*). Nevertheless, recently bacteria have also been widely explored. They grow faster than fungi, therefore the biosynthesis of the cellulolytic enzymes can be more efficient. Frequently mentioned in the literature are *Bacillus* spp., *Geobacillus* spp., *Caldibacillus* spp., *Acidothermus* spp., *Caldocellum* spp. and *Clostridium* spp. (*21*, *22*). They are isolated from manifold environments. For instance, *Geobacillus thermodenitrificans*, *Bacillus licheniformis* and *Bacillus aerius* were isolated from Tattapani hot spring sediment (Himalayas) after incubation at 60 °C (*23*). Tropical mangrove soil originating from Malaysia was a source of cellulose-degrading bacteria belonging to *Anoxybacillus* sp., *Paenibacillus dendritiformis* and *Bacillus subtilis* (*24*). Compost and agricultural waste are a rich source of thermophilic cellulolytic bacteria. In these environments commonly occurring microorganisms are aerobic bacteria, belonging to the genus *Bacillus* and anaerobic *Clostridium* (*25*, *26*).

The lignocellulosic raw materials can be used in numerous biotechnological processes. This issue is the mainstream of the circular economy and it is the reason why we undertook to search for new solutions, enabling hydrolysis of plant biomass. The first step that needs to be taken towards developing new effective technology is the isolation of bacteria capable of cellulose hydrolysis at elevated temperature, exceeding 50 °C. This became the subject of this study.

## MATERIALS AND METHODS

### Isolation and preliminary screening of thermophilic bacteria

Solid samples were harvested from fermenting hay (1 g) (Zgierz, Poland, 51°51'24.8"N 19°23'42.9"E) and extracted during 60 min of mixing, in 100 mL of phosphate-buffered saline (PBS; Chempur, Gliwice, Poland) with the addition of 1 mL of polysorbate 80 (Biomaxima, Lublin, Poland). Liquid samples, containing rotting plant fragments, derived from geothermal karst spring (64°18'34.7"N 20°18'12.6"W), hot spring (64°00'34.3"N 21°11'04.9"W) and vicinity of geothermal pond Blue Lagoon (63°52'50.5"N 22°26'52.4"W) (all originating from Iceland) were used directly. Screening of thermophilic bacteria was performed using serial dilution and spread plate technique on nutrient Gelzan™ medium. It was prepared by solidification of nutrient broth (BTL, Lodz, Poland) with the addition of gellan gum 8 g/L (Gelzan™; Sigma-Aldrich, Merck, St. Louis, MO, USA) and MgSO_4_ 1 g/L (Chempur, Piekary Slaskie, Poland). Single colonies were picked after 48 h of incubation at 50 or 60 °C. Pure cultures of isolated bacteria were kept frozen at -80 °C in glycerol stocks.

### Characterisation of morphology of the isolated bacteria

Bacteria were cultured for 24 h on nutrient Gelzan^TM^ medium at the temperature of their isolation. The morphology was observed after Gram staining under the light microscope (BX63; Olympus, Tokyo, Japan) at the magnification of 1000×.

### Effect of temperature on bacterial growth

Bacterial strains were activated at 50 °C for 24 h in 20 mL of nutrient broth (BTL). The precultures were centrifuged for 10 min at 1731×*g* and 22 °C (3-18KS; Sigma-Aldrich, Merck, Osterode am Harz, Germany), and the biomass was suspended in 9 mL of sterile saline to obtain a cell amount corresponding to 0.50±0.05 on the McFarland scale or approx. 1.5·10^8^ CFU/mL (DEN-1B densitometer, Biosan, Riga, Latvia). The next step was performed in microplates and triplicated. The wells containing 170 µL of nutrient broth were inoculated with 30 µL of standardised suspension of microorganisms. The cultures were incubated at 4, 20, 25, 30, 37, 45, 50, 55, 60, 65 and 70 °C. After 24 h, the changes in the absorbance (*λ*=600 nm) were measured regarding the control sample which contained non-inoculated nutrient broth (Multiscan GO spectrophotometer; Thermo Fisher Scientific, Waltham, MA, USA).

### Comparison of bacterial growth at 50 °C

To determine the ability to grow at 50 °C the BioLector^®^ microbioreactor system (m2p-labs GmbH, Baesweiler, Germany) was used. Strains were activated overnight in 10 mL of nutrient broth at the temperature of isolation. The microplate wells containing 1250 µL nutrient broth were inoculated by 125 µL of precultures prepared as described previously. Incubation was carried out at 50 °C with shaking frequency 1000 rpm. The changes in biomass concentration expressed as scattered light intensity (*λ*=620 nm) were measured online every 15 min for 48 h starting from the moment of inoculation, using a built-in BioLector® detector. Growth curves were prepared using Microsoft Excel 2010 (*27*).

### Determination of the activity of cellulase-producing bacteria based on carboxymethylcellulose (CMCase)

Cellulase-producing bacteria were screened on carboxymethyl cellulose (CMC) agar plates containing: low-viscosity carboxymethyl cellulose sodium salt 2 g/L (Glentham Life Sciences, Corsham, UK), NaNO_3_ 2 g/L, K_2_HPO_4_ 1 g/L, MgSO_4_^.^7H_2_O 0.5 g/L, KCl 0.5 g/L (all from Chempur, Piekary Slaskie), peptone 0.2 g/L (BTL) and agar 2 g/L (VWR; Avantor, Radnor, PA, USA) (*28*). Strains were activated during 24 h of cultivation at 50 °C in nutrient broth. Then, the microorganisms were plated onto CMC agar and incubated at 50 °C for 2 days. Resulting colonies were picked up and transferred onto a new CMC agar to ensure that bacterial growth was a result of CMC degradation. After 3 days of incubation at 50 °C, the zones surrounding the colonies were visualised by Congo red (Carl-Roth GmbH & Co. KG, Karlsruhe, Germany) staining (*29*). To compare the capability of CMC degradation, hydrolysis capacity was calculated. The hydrolysis capacity is defined as the ratio of the diameter of clearing zone around the colony and the colony diameter (*30*).

### Test of bacterial enzymatic activity using API® ZYM

Bacteria were activated for 24 h at 50 °C in nutrient broth. The cultures were centrifuged for 10 min at 1731×*g* and 22 °C (3-18KS; Sigma-Aldrich, Merck), and the biomass was suspended in sterile saline to obtain a cell amount corresponding to 5-6 on the McFarland scale (approx. 1.5-1.8·10^9^ CFU/mL; DEN-1B Densitometer). API® ZYM tests (BioMerieux, Marcy-l'Étoile, France) were performed following the manufacturer's instructions. However, due to the specificity of the microorganisms, the incubation temperature was raised to 50 °C.

### Determination of oxidase, aminopeptidase and catalase activities

Microorganisms were activated on the nutrient Gelzan^TM^ medium as described earlier. The activities of the cytochrome oxidase and l-alanine aminopeptidase were detected with Bactident® Oxidase (Merck, Darmstadt, Germany) and Bactident® Aminopeptidase (Merck) test strips respectively. The tests were performed according to the manufacturer’s recommendations. The catalase activity was determined as follows: the bacterial biomass was placed in the microscope slide and a drop of 5% hydrogen peroxide (Chempur, Piekary Slaskie) was applied on the biomass. Catalase activity was confirmed based on O_2_ formation.

### Identification of the selected bacteria

Total genomic DNA was extracted and purified using NucleoSpin® microbial DNA kit (Macherey-Nagel GmbH & Co. KG, Düren, Germany) according to manufacturer instructions. DNA yield and purity were assessed by spectrophotometric method (NanoVue Plus^TM^, Biochrom, Cambridge, UK). The 16S rRNA gene was amplified with the universal primers 27F: 5’-AGAGTTTGATCCTGGCTCAG-3’, 1492R: 5’-TACGGTACCTTGTTACGACTT-3’. The PCR reaction mixture contained 4 µL of the DNA template, 1 µL of each primer at a concentration of 10 µM, 25 µL PrimeSTAR® Max DNA Polymerase (Takara, Kyoto, Japan) and 25 µL water. The polymerase chain reaction (PCR) was carried out in 30 cycles according to the following program: 10 s denaturation at 98 °C, 10 s annealing at 55 °C, and 10 s extension at 72 °C. The presence of the appropriate-size amplicons of each isolate was confirmed by agarose gel electrophoresis. The PCR products were sequenced by external service (Genomed, Warsaw, Poland). The sequences were analysed with Chromas (*31*) and Decipher (*32*) open source software, aligned and compared with the deposited sequences in GenBank (*33*).

## RESULTS AND DISCUSSION

### Characterisation of isolated bacteria and evaluation of CMCase activity

**Fig. 1:**
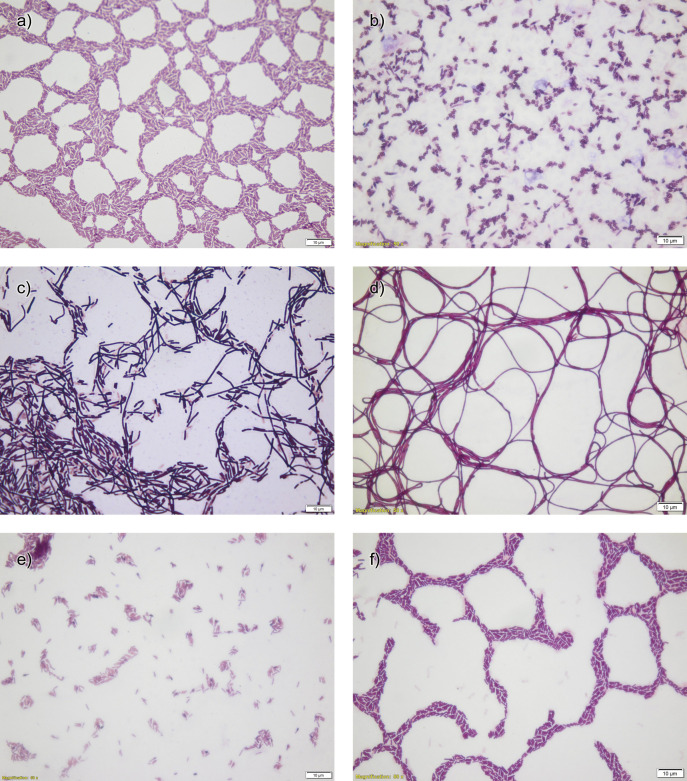
An exemplary morphology of isolated bacteria at 1000× magnification: a) BWO8, b) BGR9, c) BBLN3, d) BSO7, e) BSO14, and f) BSO8

Thermophilic bacteria described in this study were isolated from geothermal karst spring, hot spring, geothermal pond and fermenting hay. Most of the strains were obtained after incubation at 50 °C and only seven grew well at a higher temperature (Table 1). The vast majority of isolated bacteria were Gram-positive and rod-shaped. The relatively small number of them (BSO4, BSO6 and BSO8) exhibited alternative construction of the cell wall. Examples of the morphological differences are presented in Fig. 1. Similar properties are often shown by other microorganisms isolated from high-temperature environments (*34*, *35*).

**Table 1:** Characterisation of thermophilic bacteria isolated from various environments and evaluation of their cellulase activity

Source	Strain	Isolation temperature/ °C	Morphology	*d*(clearing zone)/mm		Hydrolysis capacity *d*(clearing zone)/ *d*(colony)
Geothermal karst spring (Iceland)	BWO1	50	Gram-positive, rod-shaped	43.4±0.9		1.05
	BWO2	50	Gram-positive, rod-shaped	ND		-
	BWO3	50	Gram-positive, rod-shaped	ND		-
	BWO4	50	Gram-positive, rod-shaped	ND		-
	BWO5	50	Gram-positive, rod-shaped	48.0±1.4		1.08
	BWO6	60	Gram-positive, rod-shaped	ND	-	
	BWO7	60	Gram-positive, rod-shaped	35.4±2.1		1.18
	BWO8	60	Gram-positive, rod-shaped	ND		-
Hot spring (Iceland)	BGR1	50	Gram-positive, rod-shaped	18.7±0.8		1.09
	BGR2	60	Gram-positive, rod-shaped	35.4±1.4		1.14
	BGR3	50	Gram-positive, rod-shaped	44.6±1.9		1.04
	BGR4	50	Gram-positive, rod-shaped	44.4±0.3		1.06
	BGR5	50	Gram-positive, rod-shaped	44.7±1.1		1.09
	BGR6	50	Gram-positive, rod-shaped	38.1±0.6		1.01
	BGR7	50	Gram-positive, rod-shaped	37.8±0.8		1.01
	BGR8	60	Gram-positive, rod-shaped	ND		-
	BGR9	60	Gram-positive, rod-shaped	ND		-
Geothermal pond (Blue Lagoon, Iceland)	BBLN1	50	Gram-positive, rod-shaped	24.2±4.5		2.74
	BBLN2	50	Gram-positive, rod-shaped	21.1±0.2		1.12
	BBLN3	50	Gram-positive, rod-shaped	32.1±1.6		1.17
	BBLN4	50	Gram-positive, rod-shaped	36.8±1.0		1.08
	BBLN5	50	Gram-positive, rod-shaped	39.7±1.2		1.04
	BBLN6	50	Gram-positive, rod-shaped	26.9±2,3		1.03
	BBLN7	60	Gram-positive, rod-shaped	ND		-
Fermenting hay (Zgierz, Poland)	BSO1	50	Gram-positive, rod-shaped	35.6±0.9		1.15
	BSO2	50	Gram-positive, rod-shaped	NG		-
	BSO3	50	Gram-positive, rod-shaped	ND		-
	BSO4	50	Gram-negative, rod-shaped	NG		-
	BSO5	50	Gram-positive, rod-shaped	NG		-
	BSO6	50	Gram-negative, rod-shaped	12.7±2.0		1.62
	BSO7	50	Gram-positive, rod-shaped	29.6±1.5		1.23
	BSO8	50	Gram-negative, rod-shaped	ND		-
	BSO9	50	Gram-positive, rod-shaped	32.0±0.6		1.19
	BSO10	50	Gram-positive, rod-shaped	25.9±1.2		1.30
	BSO11	50	Gram-positive, rod-shaped	41.8±1.0		1.09
	BSO12	50	Gram-positive, rod-shaped	ND		-
	BSO13	50	Gram-positive, rod-shaped	28.1±1.0		1.38
	BSO14	50	Gram-positive, rod-shaped	29.3±0.2		8.02
	1BSO15	50	Gram-positive, rod-shaped	40.1±0.9		1.05
	BSO16	50	Gram-positive, rod-shaped	32.9±0.8		1.07

ND=clearing zone was not observed, NG=strain did not grow

The bacteria showed different capability of carboxymethyl cellulose utilization (Table 1). Strains BSO2, BSO4 and BSO5 were not able to grow under the described cultivation conditions, whereas BWO2, BWO3, BWO4, BWO6, BWO8, BGR8, BGR9, BBLN7, BSO3, BSO8 and BSO12 formed colonies, but the clearing zones surrounding them were not observed. Nevertheless, most of the isolated strains utilized carboxymethyl cellulose. In the cultures of BWO1, BWO5, BGR3, BGR4, BGR5, BSO11 and BSO15, the clearing zones were larger than 40 mm in diameter. However, the most important parameter is the hydrolysis capacity. The ratio of the diameter of the clearing zone around the colony and the colony diameter allowed us to compare the cellulolytic activities and to single out the most appropriate strains. The hydrolysis capacity in most cases was approx. 1. The following strains showed favourable properties: BBLN1, BSO6, BSO10 and BSO13, with the hydrolysis capacities of 2.74, 1.62, 1.30 and 1.38 respectively (Table 1). However, its highest value was observed in the culture of BSO14 and reached 8.02.

### Effect of temperature on the growth of isolated bacteria

The bacterial growth was evaluated in the temperature range of 4-70 °C (Table 2). For most of the isolated strains, the minimum was 25 °C and the maximum did not exceed 60 °C. Only BWO3, BWO4 and BGR8 grew at a higher minimum temperature, *i.e.* 30, 37 and 45 °C, respectively. Moreover, similar to strain BGR9, BGR8 was able to grow even at 65 °C. Although the maximum growth temperatures are high, only a few strains can be classified as moderate thermophiles. Assuming that the optimal growth temperature of thermophilic bacteria cannot be lower than 50 °C, only BWO3, BWO4, BWO6, BWO7, BWO8, BGR1, BGR2, BGR8, BGR9, BBLN1, BBLN7, BSO6, BSO8 and BSO11 can be included in this group. These strains are particularly interesting for our further studies.

**Table 2:** Growth temperatures of isolated bacteria

Strain	Growth temperature range/°C	Optimal growth temperature/°C
BWO1	25-60	37
BWO2	25-50	37
BWO3	30-60	50
BWO4	37-60	50
BWO5	25-50	37
BWO6	25-60	50
BWO7	25-60	50
BWO8	25-60	50
BGR1	20-60	50
BGR2	20-60	50
BGR3	25-60	37
BGR4	25-50	37
BGR5	25-60	37
BGR6	25-50	37
BGR7	20-50	37
BGR8	45-65	50
BGR9	20-65	55
BBLN1	25-50	60
BBLN2	25-50	30
BBLN3	25-60	37
BBLN4	20-60	30
BBLN5	25-60	37
BBLN6	20-60	45
BBLN7	25-60	50
BSO1	25-60	37
BSO2	20-60	45
BSO3	25-60	37
BSO4	20-50	37
BSO5	25-45	37
BSO6	25-55	50
BSO7	25-60	37
BSO8	25-55	50
BSO9	25-60	30
BSO10	25-60	37
BSO11	25-60	55
BSO12	20-50	30
BSO13	25-60	37
BSO14	25-60	45
BSO15	20-60	30
BSO16	20-60	37

### Determination of bacterial growth at 50 °C using the BioLector® microbioreactor

**Fig. 2:**
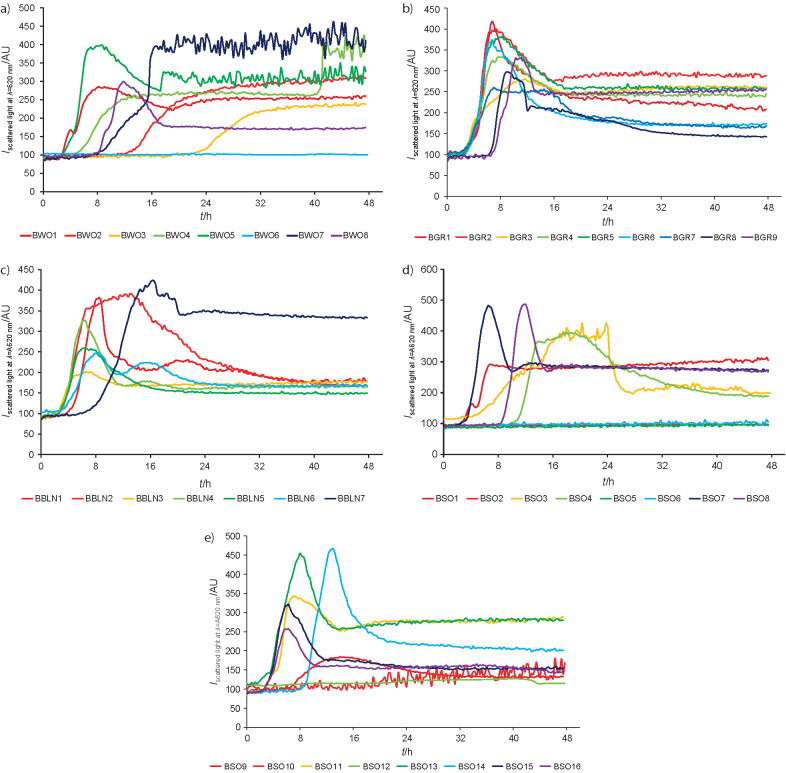
Comparison of growth of bacteria isolated from: a) geothermal karst spring, b) hot spring, c) geothermal pond, and d) and e) fermenting hay (BSO1-8 and BSO9-16 respectively) during 48 h of incubation at 50 °C in the BioLector® bioreactor

The results presented previously show that temperature is an important factor affecting bacterial cell growth. The BioLector® microbioreactor allows us to compare the cell densities in bacterial cultures after a specified incubation time and to analyse the growth phases. Based on the changes in light scattering, we tried to find the strains capable of fast and stable growth at 50 °C (Fig. 2).

The vast majority of isolated strains grew at 50 °C. However, BWO6, BSO2, BSO5, BSO6 and BSO12 did not proliferate under the described cultivation conditions (Fig. 2a, Fig. 2d and Fig. 2e). This fact is surprising since the previously determined maximum growth temperature was lower than 50 °C only for BSO5 (Table 2). We observed differences also in the length of the lag phases. In a significant number of the bacterial cultures, it lasted less than 8 h. Quite different were the cultures of strains BWO2, BWO3, BWO7, BWO8, BSO4, BSO8 and BSO14 (Fig. 2a, Fig. 2d and Fig. 2e). Their adaptation phase lasted at least 8 h, and even up to 20 h, as in the case of BWO3 strain (Fig. 2a). The scattered light intensities show that the exponential phases were usually short and intensive. However, in a few cases: BWO2, BWO3, BBLN7, BSO3 and BSO9, the logarithmic growth curve was prolonged, showing first a gentle and then a low increase in biomass concentration (Fig. 2a, Fig. 2c, Fig. 2d and Fig. 2e). A significant number of tested microorganisms did not maintain stationary phase at 50 °C. The scattered light intensities decreased rapidly immediately after reaching the maximum. Moreover, the experiment carried out in the BioLector® bioreactor allowed us to observe the phenomenon of diauxie in the cultures of the BWO4 and BWO7 strains (Fig. 2a). This may indicate adaptations of that microorganism to the demanding environmental conditions. The classic examples of a stationary growth phase can be observed only in the cultures of BWO2, BWO3 and BSO1 (Fig. 2a and Fig. 2d).

### Enzymatic activity of isolated bacteria

The bacterial strains isolated from extreme environments demonstrated a large variety of enzymatic activities (Table 3).

**Table 3:** Biotyping of the isolated bacteria based on their enzymatic activities

Strain	Enzymatic activity																						
Oxidase	Catalase	l-alanine aminopeptidase	Alkaline phosphatase	Esterase (C4)	Esterase lipase (C8)	Lipase (C14)	Leucine arylamidase	Valine arylamidase	Cystine arylamidase	Trypsin	α-chymotrypsin	Acid phosphatase	Naphthol-AS-BI-phosphohydrolase	α-galactosidase	β-galactosidase	β-glucuronidase	α-glucosidase	β-glucosidase	*N*-acetyl-β-glucosaminidase	α-mannosidase	α-fructosidase	Control
			API® ZYM																			
BWO1	+	+	-	+	+	+	-	+/-	-	-	-	+	+	-	-	+	-	+	+	-	-	-	-
BWO2	+	+	-	+/-	+	+	-	+	+/-	+/-	-	-	+/-	+/-	+	+	-	+/-	-	-	-	-	-
BWO3	+	+	-	+	+	+	-	+/-	-	+/-	-	-	+	+	+	+	-	-	+	-	+	-	-
BWO4	+	+	-	-	+	+	-	+	+/-	+	-	+/-	-	-	+	+	-	+	+	-	+	-	-
BWO5	+	-	-	+	+	+	-	-	-	-	-	+/-	+	+	-	+	-	+/-	+	-	-	-	-
BWO6	+	+	-	+	+	+	+	+	+	+	-	-	+	-	-	-	-	-	-	-	-	-	-
BWO7	+	+	-	+	+	+	-	-	-	-	-	-	-	+/-	+	+	-	+	+	-	-	-	-
BWO8	+	-	-	-	+	+	-	+	-	-	-	-	-	-	+	+	-	+	+/-	-	-	-	-
BGR1	+	-	-	+	+	+	-	-	-	-	-	+	+	+/-	-	+	-	+	+	-	-	-	-
BGR2	+	+	-	+	+	+/-	-	+	-	-	-	+	+	+	+/-	+	-	+	+	-	-	-	-
BGR3	+	+	-	+	+	+/-	-	-	-	-	-	+	+	+	-	+	-	+	+	-	-	-	-
BGR4	+	+	-	+	+/-	-	-	+/-	-	+	-	+	+	+/-	-	+	-	+/-	+/-	-	-	-	-
BGR5	+	+	-	+	+/-	-	-	-	-	-	-	+/-	+	+	-	+	-	+/-	-	-	-	-	-
BGR6	+	+	-	+	+	-	-	-	-	-	-	+	+	+	-	+	-	+/-	+/-	-	-	-	-
BGR7	+	+	-	+	+	+	-	+/-	-	-	-	+	+	+	+	+	-	+/-	+	-	-	-	-
BGR8	+	+	-	+/-	+	+	+	-	-	+/-	+	-	-	-	-	-	-	-	-	-	-	-	-
BGR9	+	-	-	+	+	+/-	-	+	-	-	-	-	+	+/-	-	-	-	-	-	-	-	-	-
BBLN1	+	+	-	+	+	+	-	+	+	-	-	+/-	+	+	+/-	+/-	-	+/-	+	-	-	-	-
BBLN2	+	+	-	+	+	+	-	+/-	-	-	-	+/-	+	+	+	+	-	+	+	-	-	-	-
BBLN3	+	+	-	+	+	+	-	+	-	-	-	+	+	+/-	+/-	+	-	+	+	-	-	-	-
BBLN4	+	+	-	+	+	+	-	-	+/-	-	-	+	+	-	-	+	-	+/-	+	-	-	-	-
BBLN5	+	+	-	+	+	+	-	-	-	-	-	+	+	+	-	+/-	-	+	+	-	-	-	-
BBLN6	+	+	-	+	+	+	-	+	+/-	+/-	-	+	+	+/-	-	+	-	+/-	+	-	-	-	-
BBLN7	+	+	-	-	+	+	-	+	-	+/-	-	-	-	-	+	+	-	+	+	-	+/-	-	-
BSO1	+	+	-	+	+	+	-	+/-	+/-	-	-	+	+	+/-	+/-	+	-	+	+	-	-	-	-
BSO2	-	-	-	+	+	+	-	-	-	-	-	-	+	-	-	-	-	-	+	-	-	-	-
BSO3	+	+	-	+	+	+	-	-	-	-	-	-	+	-	-	-	-	-	+	-	-	-	-
BSO4	+	+	+	-	+	-	-	+	-	-	-	+	+/-	+/-	-	-	-	-	-	-	-	-	-
BSO5	+	+	-	+	+	+	-	-	+/-	+/-	-	+	+	+	-	+	-	+	+	-	-	-	-
BSO6	+	-	+	+/-	+	+	-	+/-	-	-	-	-	+/-	+	-	-	-	-	-	-	-	-	-
BSO7	+	+	-	+	+	+/-	-	+	+/-	-	-	+	+	+	+/-	+	-	+	+	-	-	-	-
BSO8	+	+	+	-	+	+	-	+	-	+/-	-	+	+/-	-	-	-	-	-	-	-	-	-	-
BSO9	+	+	-	+/-	+	+/-	-	-	-	-	-	-	+	+/-	-	-	-	-	+	-	-	-	-
BSO10	+	+	-	+/-	+	+	-	-	-	-	-	+/-	+	+	-	-	-	+	+/-	-	-	-	-
BSO11	+	+	-	+	+	+	-	+/-	-	-	-	+	+	+	-	+	-	+	+	-	-	-	-
BSO12	+	+	-	+/-	+	+	-	+/-	+/-	-	-	+/-	+/-	+	-	+/-	-	+	+	-	-	-	-
BSO13	+	+	-	+	+	+/-	-	+	+/-	-	-	+	+	+	+/-	+	-	+	+	-	-	-	-
BSO14	+	+	-	-	+	+/-	-	-	-	-	-	-	-	+	+/-	+	-	-	-	-	-	-	-
BSO15	+	+	-	+	+	+/-	-	-	-	-	-	+	+	+	-	+	-	+	+	-	-	-	-
BSO16	+	+	-	+	+	+	+/-	-	+/-	+/-	-	+	+	+	-	+	-	+	+	-	-	-	-
Number of occurrences	39	34	3	34	40	36	3	23	12	11	1	27	34	30	15	30	0	29	31	0	3	0	-
Occurrence frequency	often	often	very rarely	often	often	often	very rarely	moderately often	rarely	rarely	very rarely	moderately often	often	moderately often	rarely	moderately often	absence	moderately often	often	absence	very rarely	absence	

- negative reaction, +/-moderate activity, +positive reaction; occurrence frequency based on number of positive results: often=31-40, moderately often=21-30, rarely=11-20, very rarely=1-10, absence=0; place of origin: BWO1-8=geothermal karst spring, BGR1-9=hot spring, BBLN1-7=geothermal pond, BSO1-16=fermenting hay

Analysis of basic biochemical traits, *i.e.* the ability to produce oxidase and catalase, showed that only BSO2 did not exhibit both of these activities. Thus, the mentioned strain could be anaerobic bacteria. This conclusion finds its confirmation in previous observations, which indicated that BSO2 is not able to grow in aerated cultures. Another enzymatic activity, l-alanine aminopeptidase, occurs almost exclusively in the cell walls of Gram-negative bacteria. Among the isolated strains, only BSO4, BSO6 and BSO8 exhibited this feature. The Gram-positive behaviour, also presented in these studies, is analogous. All enzyme activities evaluated by the API® ZYM test occurred with a similar frequency among microorganisms, regardless of their origin. Alkaline phosphatase, esterase (C4), esterase lipase (C8), acid phosphatase and β-glucosidase activities were found often, whereas leucine arylamidase, α-chymotrypsin, naphthol-AS-BI-phosphohydrolase, β-galactosidase and α-glucosidase occurred moderately often (Table 3). Particularly interesting are activities detected rarely or very rarely. Up to 15 strains show the activities of valine arylamidase, cystine arylamidase and α-galactosidase. Very rare lipase (C14), trypsin and α-mannosidase activities were found. The lipase (C14) was detected in BWO6, BGR8 and BSO16. Furthermore, BGR8 also contained trypsin. The presence of α-mannosidase was found in BWO3, BWO4 and BBLN7 cells. None of the tested strains contained β-glucuronidase, *N*-acetyl-β-glucosaminidase or α-fructosidase.

### Identification of bacteria

Cultures of bacteria selected for this part of the study were capable of growing at least at 37 °C and exhibited hydrolysis capacity values greater than 1.3. Strains that meet the selection criteria are BBLN1, BSO10, BSO13 and BSO14. The results of the analyses of their 16S rDNA sequences are presented in Table 4. According to the obtained results BBLN1, BSO10 and BSO13 were classified as *Bacillus licheniformis*, and BSO14 as *Paenibacillus lactis*.

**Table 4:** Identification of cellulolytic strains

Strain	Species name	GenBank accession number	Collection number
BBLN1	*Bacillus licheniformis*	MT454001	LOCK 1149
BSO10	*Bacillus licheniformis*	MT459334	LOCK 1147
BSO13	*Bacillus licheniformis*	MT459335	LOCK 1148
BSO14	*Paenibacillus lactis*	MT459409	LOCK 1150

*Paenibacillus* was distinguished from *Bacillus* group 3 based on 16S rRNA analysis (*36*). Bacteria belonging to *Paenibacillus* sp. have been repeatedly isolated from a variety of environments including fresh and salty water, soil, bentonite, plants, rhizosphere, compost, sewage, sediments and caves. Its presence was reported also in food, insect larvae and human clinical samples (*37*-*39*). The main sources of *Paenibacillus lactis* that have been described so far are soil, water, raw and ultra-high temperature (UHT) milk and a dairy farm environment (*37*, *40*-*42*). It was reported that the endospores of *P. lactis* can survive UHT processing of milk as well as industrial sterilisation (*37*). Therefore, it is not surprising that *P. lactis* BSO14 that we isolated was able to outlast inside the fermenting hay, where the highest recorded temperature reached 52 °C. However, the optimal growth temperature of BSO14 reached 45 °C and was higher than usually described for *P. lactis*, which is about 30-40 °C. Moreover, the BSO14 strain showed the ability to proliferate even at 60 °C, which has not yet been reported (*37*, *41*). *P. lactis* can utilize various carbon sources, for example, arabinose, cellobiose, fructose, d-glucose, glycogen, lactose, maltose, mannitol, mannose, melibiose, raffinose, ribose, starch, sucrose, trehalose and xylose (*37*, *41*). Although many representatives of *Paenibacillus* can degrade cellulose, so far it has not been confirmed for *P. lactis* (*43*). New strain *P. lactis* BSO14 exhibited high cellulose hydrolysis capacity. With a few exceptions, the value of this parameter was nearly 8 times higher than for other isolated strains. Based on the API® ZYM test, strain BSO14 showed also the activities of esterase (C4), esterase lipase (C8), naphthol-AS-BI-phosphohydrolase, α-galactosidase and β-galactosidase. BSO14 is a new strain with unique properties, interesting for industrial applications.

BBLN1, BSO10 and BSO13 described in this manuscript also exhibited valuable properties. The strains were identified as *Bacillus licheniformis*. Their presence is not surprising since *B. licheniformis* is one of the most prevalent *Bacillus* species that inhabits a wide variety of environments and its cellulolytic properties are well known. Among them are hot springs (*44*), compost (*45*) and mangrove soil (*46*). The microorganisms we described originate from Blue Lagoon (Iceland) and fermenting hay (Poland). The sources of their isolation are similar to those previously described. *B. licheniformis* is classified as mesophilic microorganism. *B. licheniformis* BSO10 and BSO13 grow well at 37 °C similar to the type strain *B. licheniformis* (Weigmann) Chester (*41*). Nevertheless, a significant number of the species can be assigned to the thermophile group. The optimal incubation temperature for BBLN1 is 50 °C. *B. licheniformis* BL1 (*47*) and Ad978 (*48*) have comparable properties. A broad spectrum of *B. licheniformis* enzymatic activities makes this microorganism an exceptional tool for industrial applications. Proteases, α-amylases and lipases from *B. licheniformis* have a commercial application (*49*-*51*). The cellulases discussed here are still the subject of research. Nonetheless, many publications indicate that the enzymes produced by *B. licheniformis* exhibit unique properties such as thermostability and operation at a wide range of pH (*44*, *45*, *51*). Strains BBLN1, BSO10 and BSO13 that we isolated can be a source of cellulases with equally interesting properties. However, to confirm their industrial potential, further studies are required. Other enzymatic activities of the strains are worth mentioning. *B. licheniformis* BBLN1, BSO1 and BSO13 produced specific enzymes, rare in comparison with the other examined bacteria, such as arylamidase and α-galactosidase. The strains also showed activities of alkaline phosphatase, esterase (C4), esterase lipase (C8), leucine arylamidase, α-chymotrypsin, acid phosphatase, naphthol-AS-BI-phosphohydrolase, β-galactosidase, α-glucosidase and β-glucosidase.

## CONCLUSIONS

The screening of thermotolerant microorganisms exhibiting specific features is just the beginning of industrial process development. Both *Paenibacillus lactis* BSO14 and *Bacillus licheniformis* BBLN1, BSO10 and BSO13 show desirable properties and are an excellent basis for further studies. The strains exhibited cellulose hydrolysis capacity values greater than 1.3 and were able to grow at least at 37 °C. It is worth emphasizing that this is the first report on *P. lactis* exhibiting such properties. The assessment of other enzymatic activities was performed by the API® ZYM tests. The studies proved that the isolated strains can be useful in circular bioeconomy. Due to the cellulolytic activities, our findings can be important for the biorefining industry, giving a new tool for the waste biomass hydrolysis. Screening stages described here: isolation, characterisation and identification of cellulolytic strains provide a good starting point for further research, in particular of the newly isolated *P. lactis* strain.
